# Response to comment on ‘Protein crystal lattices are dynamic assemblies: the role of conformational entropy in the protein condensed phase’

**DOI:** 10.1107/S2052252518006279

**Published:** 2018-07-01

**Authors:** Margarita Dimova, Yancho D. Devedjiev

**Affiliations:** a Department of Anesthesiology, University of Virginia, 1215 Lee Street, Charlottesville, VA 22908, USA

**Keywords:** protein crystals, crystal lattices

## Abstract

A response is given to Nespolo’s comment [*IUCrJ* (2018), **5**, https://doi.org/10.1107/S2052252518006267] about the usage of the the term ‘crystal lattice’ in Dimova & Devedjiev [*IUCrJ* (2018), **5**, 130–140].

Nespolo (2018 ▸) analyses our paper (Dimova & Devedjiev, 2018 ▸) from the point of view of crystallography that studies the asymmetric unit of the crystals. From this point of view, the crystal lattice is indeed a mathematical abstraction – a three-dimensional array of identical units. We analyze protein crystals as physical objects and demonstrate that the units in the crystal may not be identical in the same volume and at the same time. To the best of our knowledge this is a new concept that has not been proposed before.
